# Cyclic fatigue resistance of HyFlex EDM, Reciproc Blue, WaveOne Gold, and Twisted File Adaptive rotary files under different temperatures and ambient conditions

**DOI:** 10.15171/joddd.2019.026

**Published:** 2019-10-07

**Authors:** Mustafa Gündoğar, Taha Özyürek, Koray Yılmaz, Gülşah Uslu

**Affiliations:** ^1^Department of Endodontics, Faculty of Dentistry, Medipol University, İstanbul, Turkey; ^2^Department of Endodontics, Faculty of Dentistry, Istanbul Medeniyet University, İstanbul, Turkey; ^3^Department of Endodontics, Faculty of Dentistry, Hatay Mustafa Kemal University, Hatay, Turkey; ^4^Department of Endodontics, Faculty of Dentistry, Çanakkale Onsekiz Mart University, Çanakkale, Turkey

**Keywords:** Cyclic fatigue, HyFlex EDM, Reciproc Blue, Static test, WaveOne Gold

## Abstract

***Background.*** This study examined the effects of changes in temperature and environmental conditions on the cyclic fatigue resistance of Reciproc Blue, HyFlex EDM, WaveOne Gold, and Twisted File Adaptive.

***Methods.*** Forty-five Reciproc Blue (25/.08), 45 HyFlex EDM (25/.08), 45 WaveOne Gold (25/.07), and 45 Twisted File Adaptive (25/.08) files were tested for cyclic fatigue at room temperature (20°C) in air and water and at body temperature (35°C) in water. All the instruments were rotated in artificial canals made of stainless steel with an inner diameter of 1.5 mm, 60° angle of curvature and a radius of curvature of 5 mm until fracture occurred; the time to fracture was recorded in seconds, using a digital chronometer. Mann-Whitney U test was used for the statistical analysis of data, with SPSS 21.0.

***Results.*** Cyclic fatigue resistance was significantly higher in all the groups in water at an ambient temperature of 20°C compared with air and water at temperatures of 20°C and 35°C, respectively (P<0.05). The intragroup analysis revealed that HyFlex EDM had the highest cyclic fatigue resistance, followed by Reciproc Blue, WaveOne Gold, and Twisted File Adaptive in both air and water at 20°C (P<0.05). HyFlex EDM exhibited the highest cyclic fatigue resistance in water at 35°C, whereas there was no significant difference between the other groups (P>0.05).

***Conclusion.*** Within the limitations of the present study, an increase in the ambient temperature significantly decreased the cyclic fatigue resistance of the tested NiTi files.

## Introduction

The fracture of nickel-titanium (NiTi) rotary instruments during root canal treatment poses a major problem for clinicians.^1^ The prognosis of root canal treatment can be negatively affected when an instrument fractures. Various thermomechanical methods have been investigated to improve the mechanical properties of NiTi rotary instrument systems.^2^ According to the manufacturers, thermomechanical treatments improved the cyclic fatigue resistance of new-generation files when compared with that of conventional NiTi files.^3^ A NiTi alloy has two heat-dependent crystal phases (martensite and austenite). The characteristics of the metal differ greatly between the martensite and austenite phases. At temperatures higher than the transformation level, NiTi alloy has an austenite structure and is rigid, whereas it generally has a martensite structure at lower temperatures and is more flexible. When the NiTi alloy is heated, martensite transforms into austenite, which has shape memory properties.^4,5^ As compared with conventional alloys, newly developed alloys have higher transformation temperatures that are closer to body temperature. Previous studies demonstrated that files made of martensite alloy had higher fracture resistance than those made of austenite alloy.^6-10^

Reciproc (RPC; VDW, Munich, Germany), updated to Reciproc Blue (RPC Blue; VDW), and WaveOne (WO; Dentsply Sirona, Baillagues, Switzerland), updated to WaveOne Gold (WOG; Dentsply Sirona), are among the most popular reciprocating files. The cross-section, dimension, and geometry of the WOG system were modified to increase the flexibility of the file. In addition, the metallurgy was changed from M-Wire to Gold-Wire to increase the fracture resistance of the file.^11^ In the RPC Blue system, the molecular structure was modified via a new heat treatment technique to improve cyclic fatigue resistance.^12^ The HyFlex EDM (HEDM; Coltene/Whaledent, Altstätten, Switzerland) rotary file system is a new-generation single-file system, which has a continuous rotation motion. HEDM (25/.08) files are made of controlled memory alloy via heat treatment. According to the manufacturer, this heat treatment has significantly improved the mechanical properties of the files.^13^ The Twisted File Adaptive (TFA; Axis/SybronEndo, Orange, CA, USA) system consists of three files, which have both rotation and reciprocation motions, depending on the pressure applied on the file. TFA files are manufactured by twisting the raw material (alloy) in the R phase of the thermal cycle.^14^

Most cyclic fatigue studies of NiTi files have been carried out at room temperature.^3,7,8^ These do not represent clinical conditions, as NiTi rotary files are used in the root canal, which has a temperature similar to that of the body. A previous clinical study reported that the intracanal temperature during root canal treatment was approximately 35°C.^15^ To date, no studies have compared the cyclic fatigue resistance of WOG, RPC Blue, HEDM and TFA files at room and intracanal temperatures. This study examined the effects of changes in temperatures and environments on the cyclic fatigue resistance of the tested files. The null hypotheses of the present study were as follows:

1. There would be no difference between the cyclic fatigue resistance of the tested NiTi files at room temperature (20°C) and intracanal temperature (35°C).
2. There would be no difference between the cyclic fatigue resistance of the tested NiTi files in air (20°C) and water (20°C) environments.

## Methods

Forty-five RPC Blue R25 (25/.08), 45 HEDM (25/.08), 45 WOG Primary (25/.07), and 45 TFA ML1 (25/.08) files were included in the present study. Before the cyclic fatigue test, the files were examined using a stereomicroscope at ×20 magnification (Imaging Systems, Leica Ltd., Cambridge, U.K.) to detect deformation. No deformation was detected, and all the files were included in the study. The files from each system were randomly divided into three groups, with 15 files in each group. The groups were as follows.

**Group 1:** The files were tested in air at 20 °C (control group).

**Group 2:** The files were immersed in distilled water at 20°C during testing.

**Group 3:** The files were immersed in water at 35 °C during the test. The ambient temperature was controlled simultaneously by an electronic thermometer.

For the static cyclic fatigue resistance test, a stainless steel artificial canal with a 5-mm radius of curvature, 60° angle of curvature, and 1.5-mm inner diameter was used.^11^ The center of the curvature of the canal was located 5 mm coronal to the apical endpoint. In the control group, the files were lubricated using a synthetic lubricant (WD-40 Company, Milton Keynes, U.K.) to minimize friction between the canal and files and to ensure free rotation of the files within the artificial canal (in group 1). The RPC Blue files were used with a VDW Reciproc Gold (VDW) endodontic motor, mounted on a cyclic fatigue test device in the “Reciproc ALL” reciprocation program until fracture occurred. The WOG files were used with a VDW endodontic motor, mounted on a cyclic fatigue test device in the “WaveOne ALL” reciprocation program until fracture occurred. The HEDM files were used with a VDW endodontic motor, mounted on a cyclic fatigue test device at 500 rpm and 2.5 gcm^-1^ torque in rotation motion until fracture occurred. The TFA files were used with an Elements motor (Axis/SybronEndo), mounted on a cyclic fatigue test device in the “TF Adaptive” adaptive program until fracture occurred. All the files were used in the artificial canal until fracture occurred, and the time to fracture was recorded using a digital chronometer. The number of cycles until failure (NCF) was then calculated according to the formula below:

NCF = revolutions per minute (rpm) × time to fracture (sec)/60.

The lengths of the fractured (FL) parts were measured using a digital caliper. In total, 24 files (n=2/each group) were examined under a scanning electron microscope (SEM; JEOL, JSM-7001F, Tokyo, Japan) to confirm that the files fractured due to cyclic fatigue.

### Statistical Analysis 

The data were first analyzed using the Shapiro–Wilk test to verify the assumption of normality. One-way ANOVA and post hoc Tamhane tests were then conducted. The data were analyzed using SPSS 21.0 (IBM-SPSS Inc., Chicago, IL, USA). The statistical significance level was set at 5%.

## Results

The means and standard deviations of cyclic fatigue resistance values of NCF and FL of the TFA, WOG, HEDM, and RPC Blue files at different temperatures and in different environments (20°C in air, 20°C in water, and 35°C in water) are shown in Table 1. Cyclic fatigue resistance was significantly higher in all the groups in water at an ambient temperature of 20°C compared with air and water at temperatures of 20°C and 35°C, respectively (P<0.05). The intragroup analysis revealed that HEDM had the highest cyclic fatigue resistance, followed by RPC Blue, WOG, and TFA in both the air and water at 20°C (P<0.05). HEDM had the highest cyclic fatigue resistance in water at 35°C, whereas there was no significant difference between the other groups (P>0.05). There were no significant differences between the groups in the mean lengths of the fractured fragments of the files at any ambient temperature (P>0.05).

**Table 1:** The means and standard deviations (SD) of the number of cycles to fracture (NCF) and fractured fragment length (FL) of instruments in water at 20°C, 35°C and in air at 20°C

	**Air at 20°C**		**Water at 20°C**		**Water at 35°C**		
	**NCF**	**FL**	**NCF**	**FL**	**NCF**	**FL**	**P -value**
**TF Adaptive**	1242 ± 149 ^ax^	5.69 ± 0.54	3067 ± 429 ^ay^	5.58 ± 0.49	1139 ± 136 ^ax^	5.58 ± 0. 57	<0.05
**WaveOne Gold**	1701 ± 214 ^bx^	5.62 ± 0.52	4626 ± 565 ^by^	5.60 ± 0.57	1206 ± 148 ^az^	5.58 ± 0.49	<0.05
**HyFlex EDM**	3289 ± 427 ^cx^	5.72 ± 0.50	9847 ± 1378 ^cy^	5.72 ± 0.56	1812 ± 198 ^bz^	5.69 ± 0.55	<0.05
**Reciproc Blue**	2748 ± 412 ^dx^	5.62 ± 0.55	7914 ± 1266 ^dy^	5.63 ± 0.48	1349 ± 161 ^az^	5.61 ± 0.53	<0.05
**P -value**	<0.05	>0.05	<0.05	>0.05	<0.05	>0.05	

* Different superscript letter indicates statistically significant at 5% level (^a,b,c,d^; for intra-group comparison; ^x,y,z^; for inter-group comparison)

**Figure 1:**
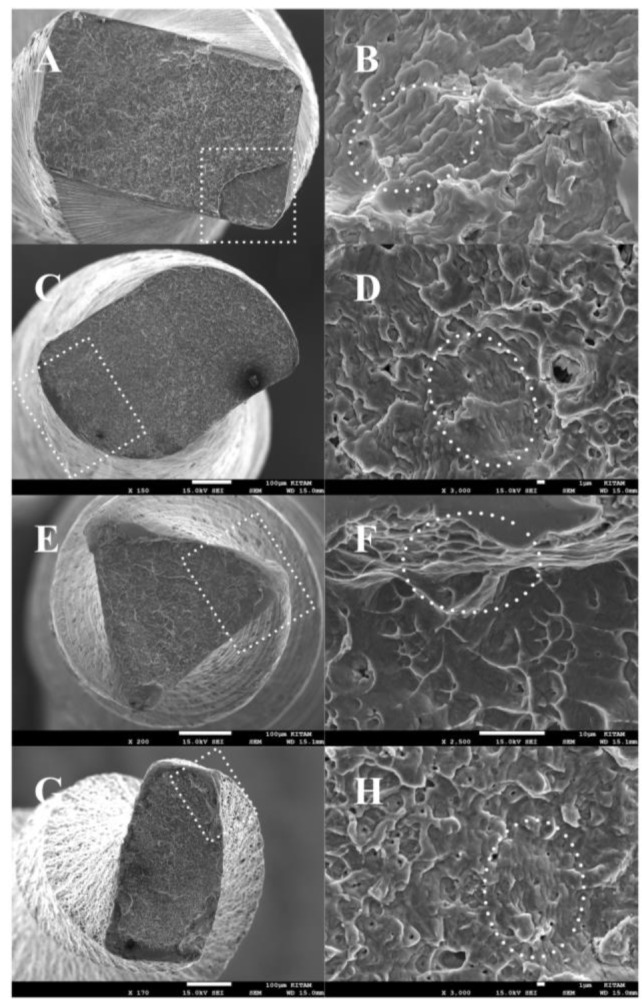
Scanning electron microscopic views of the HEDM, WOG, RPC Blue, and TFA files after cyclic fatigue testing. The fractured surface views of (A) WOG, (C) RPC Blue, (E) TFA, and (G) HEDM Blue and a high-magnification view of (B) WOG, (D) RPC Blue, (F) TFA, and (H) HEDM Blue instruments. The crack initiation origin and overload zone (A, C, E, G) and typical fatigue striations of cyclic fatigue (B, D, F, G) (white dottled line).

The SEM analysis of the fractured cross-sectional surfaces revealed typical features, including crack origins, fatigue zones, and an overload (i.e., fast fracture) zone, of cyclic failure (Figure 1).

## Discussion

The super-elastic character of NiTi files is due to the transformation of the crystal structure of martensite in response to stress. This transformation is reversible, with a phase transition between austenite and martensite. Thus, the temperature at which the transformation between the two phases occurs plays a vital role in the mechanical properties of NiTi files.^2^ Almost all the previous studies on the cyclic fatigue in the literature have been carried out at room temperature. However, in the clinical setting, NiTi rotary file systems are used in root canals. According to various studies, the intracanal temperature level varies from 31°C to 33.5°C.^15,16^ Thus, the present study aimed to examine the effects of different temperatures and environments on the cyclic fatigue resistance of various NiTi files.

In the present study, the cyclic fatigue resistance of all the files tested in 20°C distilled water significantly increased as compared with that of files tested in 35°C distilled water. Thus, the first null hypothesis was refuted. Similar to the results of the present study, de Vasconcelos et al^17^ reported that the cyclic fatigue resistance of HyFlex CM (Coltene/Whaledent), Vortex Blue (Dentsply Sirona), TRUShape (Dentsply Sirona), and ProTaper Universal (Dentsply Sirona) files significantly decreased in distilled water at 37°C as compared with distilled water at 20°C. Moreover, Jamleh et al^18^ reported a statistically significant decrease in cyclic fatigue resistance of NiTi instruments at 37°C and 50°C when compared with 10°C. Plotino et al^19^ reported that the body temperature (35°C) did not influence the cyclic fatigue of ProTaper Gold (Dentsply Sirona) files, whereas the cyclic fatigue resistance of ProTaper Universal files decreased significantly at this temperature. The researchers attributed this result to the transformation temperature of ProTaper Gold files, which is higher than that of the body temperature. Dosanjh et al^20^ reported that the cyclic fatigue resistance of all the EdgeFile (EdgeEndo, Albuquerque, NM, USA), Vortex Blue and EndoSequence (Brasseler USA, Savannah, GA, USA) NiTi files was lower at 37°C and 60°C when compared with 3°C and 10°C. Grande et al^21^ demonstrated that cooling the files from room temperature (20°C) down to -20°C had a positive effect on the cyclic fatigue resistance of NiTi files. NiTi alloys are generally in the austenite phase. Files in this phase are stiffer and have a more fragile structure than files in the martensite phase. At temperatures below the transformation temperature, files are in the martensite phase and have a more flexible structure than files in the austenite phase.^5,22,23^ The decrease in cyclic fatigue resistance of the NiTi files tested in the present study at higher temperatures might be attributed to transformation into the austenite phase. Previous studies showed that micro-crack formation and crack propagation rates were lower in the martensite phase than in the austenite phase.^24^ The martensitic structure of the files at room temperature (lower than transformation temperature) might explain their enhanced cyclic fatigue resistance.

According to the findings of the present study, when compared with other NiTi file systems, the HEDM NiTi files showed higher cyclic fatigue resistance under all the temperature and environmental conditions, consistent with similar previous studies.^25-28^ Many methods, including heat treatment, have been developed to increase the flexibility of NiTi files and improve their cyclic fatigue resistance.^29-31^ A differential scanning calorimetry study showed that the transformation temperatures of files subjected to heat treatments increased, approaching that of the body temperature.^29^ The higher cyclic fatigue resistance of HEDM files can be attributed to their transformation temperature, which is higher than that of the other files tested. Moreover, the electro-discharge machining procedure implemented during the production of these files might have contributed to the cyclic fatigue of the files.

According to the results of the present study, RPC Blue exhibited the best cyclic fatigue resistance in 20°C air and distilled water environments, excluding the HEDM, followed by WOG and TFA, whereas there was no significant difference in the cyclic fatigue resistance of the files in a 35°C water environment. Previous studies reported that reciprocation motion increased the cyclic fatigue life of NiTi files when compared with continuous rotation motion.^32-34^ Similar to the results of the present study, Gündoğar and Özyürek^25^ reported that RPC Blue exhibited significantly higher cyclic fatigue resistance than WOG files at room temperature. The difference between the cyclic fatigue resistance of the files in terms of increases in temperature might be due to their different transformation temperatures.

According to the results of the current study, the cyclic fatigue resistance of all the tested NiTi files was significantly higher at 20°C in distilled water than at 20°C in air. Thus, the second null hypothesis of this study was rejected. Similarly, de Vasconcelos et al^17^ reported that the cyclic fatigue resistance of HyFlex CM, Vortex Blue, TRUShape, and ProTaper Universal files was significantly higher in distilled water at 20°C than at 20°C in an air environment. The lower cyclic fatigue resistance shown by the NiTi files might be due to the local increases in temperatures.^35^

In this study, the mean lengths of the fractured segments in all the groups did not show any significant differences. The fractured length of each ﬁle occurred at the center of curvature or just below this point, which conﬁrms that the instruments were positioned in a precise trajectory.

It is difficult to draw comparisons between different brands because of differences in designs, cross-sectional areas and the alloys used. Moreover, in the clinical use, different irrigation solutions and forces applied might affect the cyclic fatigue resistance of files. Thus, caution should be exercised in extrapolating the results of the present study to the clinical setting. Moreover, for future studies, the intracanal temperature should be taken into account, especially while testing the cyclic fatigue resistance of heat-treated NiTi files.

## Conclusion

Within the limitations of this in vitro study, the following conclusions can be drawn:

1. Distilled water at an ambient temperature significantly increased the cyclic fatigue resistance of the tested NiTi files as compared with the same temperature in air.
2. An increase in the ambient temperature significantly decreased the cyclic fatigue resistance of the tested NiTi files.

## Acknowledgement

The authors deny any conflicts of interest related to this study.

## Authors’ Contributions

MG, KY, GU and TÖ conceptualized the study. GU and TÖ were responsible for collection of data. MG and TÖ analyzed the results. KY, GU and TÖ were responsible for funding acquisition. MG and TÖ reviewed the literature. KY, GU and TÖ designed the methodology. MG and TÖ were responsible for the project administration. KY, GU and TÖ were responsible for the resources. MG and TÖ supervised the study procedures. MG and TÖ validated the study. KY and GU prepared the initial draft. TÖ reviewed and revised the draft.

## Funding

Not applicable.

## Competing Interests

The authors declare no competing interests with regards to the authorship and/or publication of this article.

## Ethics Approval

Not applicable
